# CREB fusion–associated epithelioid mesenchymal neoplasms of the female adnexa: three cases documenting a novel location of an emerging entity and further highlighting an ambiguous misleading immunophenotype

**DOI:** 10.1007/s00428-023-03546-1

**Published:** 2023-04-25

**Authors:** Alexis Trecourt, Nicolas Macagno, Carine Ngo, Charles-André Philip, Jonathan Lopez, Joana Ferreira, Catarina Alves-Vale, Isabelle Ray-Coquard, Catherine Genestie, Abbas Agaimy, Mojgan Devouassoux-Shisheboran

**Affiliations:** 1 Department of Pathology – Hospices Civils de Lyon - Centre Hospitalier Lyon Sud, University Claude Bernard Lyon I, 69495 Pierre Bénite, Lyon, France; 2 Department of Pathology, Aix Marseille University, INSERM, APHM MMG, UMR1251, Marmara Institute, La Timone Hospital, Marseille, France; 3 Department of Pathology, Gustave Roussy, 94805 Villejuif, France; 4 Department of Gynecology, Hospices Civils de Lyon –Hôpital de la Croix Rousse, 69004 Lyon, France; 5 Department of Biochemistry and Molecular Biology, Hospices Civils de Lyon - Centre Hospitalier Lyon Sud, 69495 Pierre Bénite, Lyon, France; 6 Serviço de Anatomia Patologica, Instituto Português de Oncologia de Lisboa Francisco Gentil Rua Prof.Lima Basto, 1099-023 Lisboa, Portugal; 7 Department of Pathology, CUF Descobertas Hospital, CUF Oncologia, 1998-018 Lisbon, Portugal; 8 Department of Medical Oncology –Centre Léon Bérard, University Claude Bernard Lyon I, 69008 Lyon, France; 9 Institute of Pathology, Friedrich-Alexander-University Erlangen-Nürnberg, University Hospital, Erlangen, Germany

**Keywords:** Epithelioid tumor, Ovary, *EWSR1::ATF1* fusion, Angiomatoid fibrous histiocytoma, Female adnexa, CREB, CREM

## Abstract

*EWSR1/FUS-CREB*-rearranged mesenchymal neoplasms are an emerging heterogeneous group of soft tissue tumors that encompasses low-grade lesions (angiomatoid fibrous histiocytoma/AFH) and a group of predominantly intra-abdominal aggressive sarcomas with epithelioid morphology and frequent keratin expression. Both entities occasionally harbor *EWSR1::ATF1* fusions as alternate to the more frequent *EWSR1/FUS::CREB1/CREM* fusions. Although *EWSR1/FUS-CREB-*rearranged epithelioid malignant neoplasms have been described in diverse intra-abdominal sites, none involved the female adnexa. Herein, we describe three cases involving uterine adnexa in young females (41, 39, and 42-year-old); two associated with constitutional inflammatory symptoms. The tumors presented as a serosal surface mass of the ovary without parenchymal involvement (Case 1), as circumscribed nodule within ovarian parenchyma (Case 2), and as a periadnexal mass extending into the lateral uterine wall with lymph node metastasis (Case 3). They were composed of sheets and nests of large epithelioid cells with numerous stromal lymphocytes and plasma cells. The neoplastic cells expressed desmin and EMA, and variably WT1. One tumor expressed in addition AE1/AE3, MUC4, synaptophysin, chromogranin, and ALK. None expressed sex cord-associated markers. RNA sequencing identified *EWSR1*::*ATF1* fusions in two cases and an *EWSR1::CREM* fusion in one. Exome-based RNA capture sequencing and clustering methods showed high transcriptomic proximity of tumor 1 with soft tissue AFH. This novel subset of female adnexal neoplasms should be included in the differential diagnosis of any epithelioid neoplasm involving female adnexa. Their aberrant immunophenotype can be misleading, underlining a wide spectrum of differential diagnosis.

## Introduction

ATF1, CREB1, and CREM constitute a subfamily of the basic leucine zipper (bZIP) superfamily of transcription factors. These proteins, known as the CREB family of transcription factors, play a crucial physiological role in various cellular functions by binding to the CRE (cAMP responsive element) sequence [1, 2]. In human tumors, the genes encoding the CREB family of transcription factors are involved in gene fusion with either *EWSR1* or *FUS* as partners. *EWSR1::ATF1* and *EWSR1::CREB1* are the two most characterized CREB fusions. They have been identified in a variety of tumors of different phenotypes and locations, including clear cell sarcomas of soft tissue, malignant gastrointestinal neuroectodermal tumor (former term: clear cell sarcoma-like tumors of the gastrointestinal tract), angiomatoid fibrous histiocytoma (AFH), clear cell carcinoma of the salivary gland, clear cell odontogenic carcinoma, a rare subset of myoepithelial tumors, primary pulmonary myxoid sarcoma (in which *EWSR1::CREB1* is a signature fusion), and primary intracranial myxoid sarcomas [3, 4]. Recently, the morphologic spectrum of tumors characterized by *EWSR1/FUS::CREB* fusions has been expanded to include a distinctive intra-abdominal malignant epithelioid neoplasm of the peritoneal cavity, and intra-abdominal organs in young adults and also a subset of epithelioid mesothelioma [5–8].

Although involvement of the kidney, liver, stomach, colon, adrenal, and pancreas has been described in *EWSR1/FUS::CREB*-rearranged malignant epithelioid neoplasms, none of them presented initially as a gynecological tumor [6, 8]. Accordingly, *EWSR1/FUS::CREB* fusion neoplasms are not well known in the female genital tract pathology literature and are, hence, possibly under-recognized. To our knowledge, only one case of putative ovarian AFH harboring *EWSR1::CREB1* gene fusion has been reported so far [9]. Herein, we present three epithelioid neoplasms with *EWSR1::ATF1/CREM* gene fusions presenting as ovarian/ adnexal tumors.

## Material and methods

From 2019 to 2023, female adnexal tumors with *ESWR1/FUS::CREB* fusions were retrieved from the files of the department of pathology of Hospices Civils de Lyon and Gustave Roussy Institute (France) and the Institute of Pathology, Erlangen (Germany). In each department, one case was found (*n*=3); one was a consultation case (AA). All H&E-stained slides were reviewed by senior pathologists (MDS, CG, NM, CN, and AA). For each case, one representative block was selected for ancillary analyses.

Immunohistochemical analyses were performed using Ventana Benchmark (Ventana-Roche, Meylan France) automated immunostainers according to the manufacturer’s recommendations. However, due to the collaborative nature of the cases, immunohistochemistry techniques and panels varied among the contributing institutions. Details of the staining protocols and antibody sources are available upon request.

Targeted RNA sequencing (RNAseq) was performed on all cases. Total nucleic acid was extracted using the Maxwell 16 LEV RNA FFPE Kit on a Maxwell 16 instrument (Promega Corporation, Madison, USA) without DNAse treatment. The areas of interest were manually scraped from unstained 4-μm formalin-fixed paraffin-embedded tumor tissue sections. RNA quantification was performed using the Qubit fluorometer (Life Technologies, Carlsbad, CA, USA). Library preparation was performed from 250 ng of RNA following the Archer Fusion-Plex Protocol for Illumina (ArcherDX Inc., San Diego, CA). An in-house pan-cancer sequencing panel (RNA Seq ARCHER Panel FusionPlex_CHU_Lyon_Pan_Solid_Tumor _Sarcoma_17125-v1.0) was used. Sequencing results were analyzed using the Archer Analysis software (v6.2; ArcherDX Inc.). Case 2 was tested using the TruSight RNA Fusion panel (Illumina, Inc., San Diego, CA, USA) as described previously [10].

## Results

### Cases history

#### Case 1

A 41-year-old woman without previous illness presented to the emergency department with fever, anemia, and weight loss of 20 kg over 3 months. There was also an inflammatory syndrome with increased serum CRP (C-reactive protein) up to 300 mg/L, without detectable infectious cause. She received steroids but developed ketoacidosis that necessitated a transfer to the intensive care unit. MRI revealed a 52-mm left adnexal mass without peritoneal spread. A left salpingo-oophorectomy was performed. The patient recovered immediately after tumor resection, and her inflammatory markers/symptoms have resolved/ normalized. The patient had no additional surgery and received no adjuvant therapy. She remained well 16 months after surgery and maintained low levels of CRP on close follow-up visits.

#### Case 2

The second patient was a 39-year-old female with a history of Hodgkin lymphoma treated by chemotherapy and bone marrow transplantation 14 years earlier. She was diagnosed on follow-up with an asymptomatic ovarian mass clinically and on pelvic MRI interpreted as endometriotic cyst. Due to suspicion of malignancy at frozen section, total abdominal hysterectomy and bilateral salpingo-oophorectomy were performed. No adjuvant treatment was given. At last follow-up, she was alive without disease relapse or metastases (10 months).

#### Case 3

**Fig. 1:**
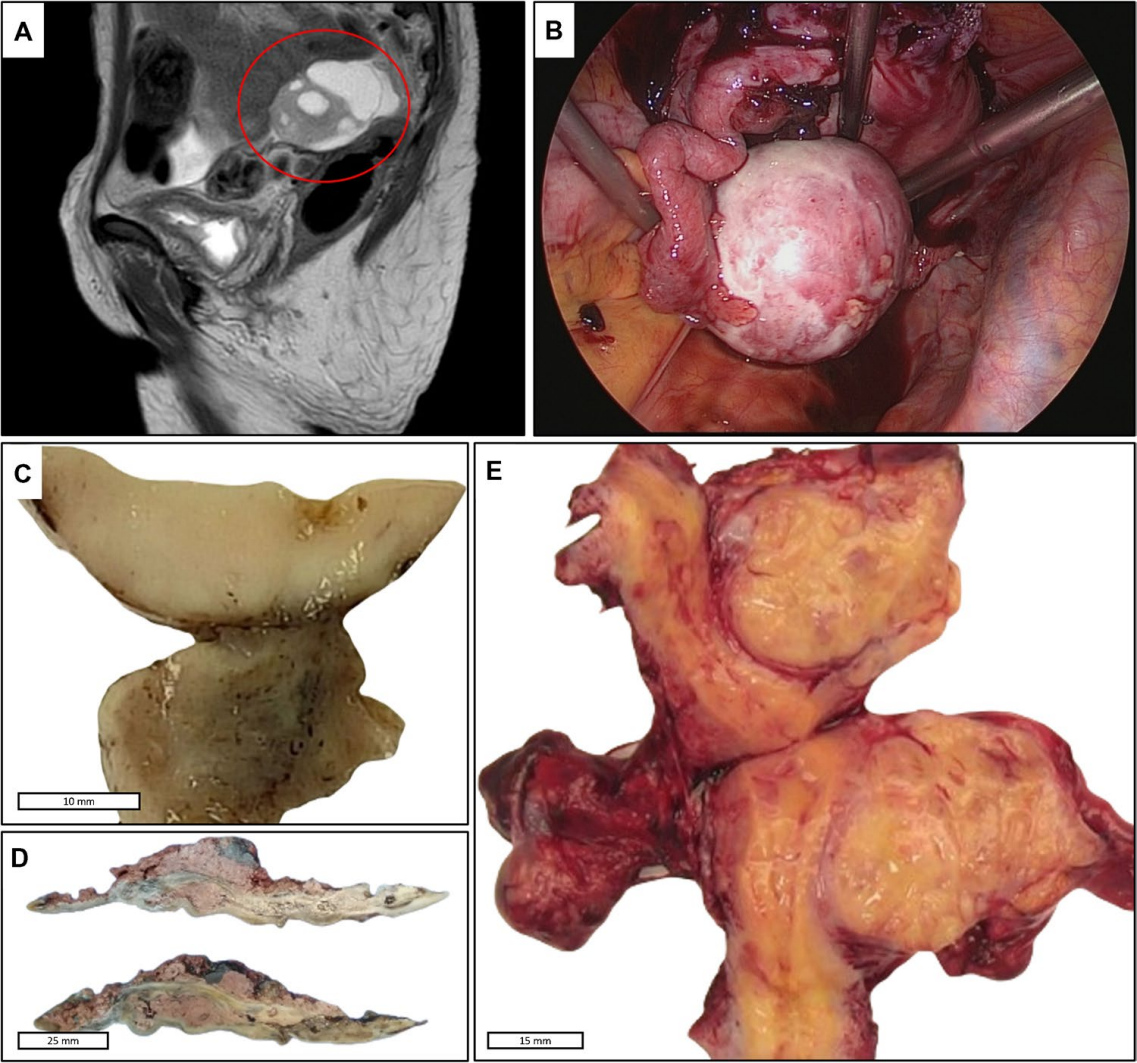
Radiological and gross findings in EWSR1/FUS-CREB-rearranged adnexal neoplasms. **A** (Case 1): sagittal T2-weighted pelvic MRI showed a 52-mm left adnexal mass (red circle) without peritoneal involvement. **B** (Case 3): coelioscopic view showing a solid periadnexal nodular mass. **C** (Case 1): gross examination revealed a solid nodule located on the surface of the ovary without infiltration of ovarian stroma. **D** (Case 2): gross examination demonstrated a hemorrhagic cystic lesion within the ovarian parenchyma reminiscent of an endometriotic cyst. **E** (Case 3): gross examination showed a 6 cm solid and yellow mass extending from the periadnexal tissue to the lateral wall of the myometrium

The third case was a 42-year-old lady who presented with uterine bleeding. MRI revealed a left latero-uterine mass of 60 mm associated with an iliac lymphadenopathy. The patient had an inflammatory syndrome with elevated CRP level. The mass was excised via coelioscopy (Fig. 1). Because of suspicion of malignancy, total hysterectomy with bilateral salpingo-oophorectomy with resection of a large iliac lymph node was performed. The patient recovered immediately after tumor resection, and inflammatory markers normalized. She received no additional therapy. She is well without recurrence after 14 months of follow-up.

### Pathological findings

Grossly (Fig. 1), the tumors measured 55 to 60 mm in maximum diameter. The cut-surfaces of periadnexal tumors were solid, tan and soft, in two cases, well circumscribed without involvement of ovarian or tubal parenchyma in Case 1 and with extension into the myometrium in Case 3. Case 2 was an ovarian mass and showed extensively hemorrhagic cut surface mimicking endometriotic cyst.

#### Histological findings (Fig. 2)

On microscopic examination, the three tumors were similar. Cases 1 and 2 were well circumscribed, surrounded by a thin fibrous capsule with lymphocytic infiltrate, while the last case showed nodular extension into the myometrium and metastasis within iliac lymph node. The neoplastic cells were epithelioid and large, arranged in nests and sheets, often in a syncytial pattern. There was no papillary or glandular architecture. Tumor cells showed a large amount of pale eosinophilic or clear cytoplasm containing round nuclei with smooth nuclear contours and vesicular chromatin. Some degree of pleomorphism was focally seen, with cells containing two or more nuclei. Mitoses were rare (0.66 mitoses/mm^2^), but few atypical mitoses were seen. Prominent chronic inflammatory cells consisting of lymphocytes and plasma cells were dispersed throughout the tumor without well-formed follicular lymphocytic aggregate. Thin collagen bundles with inflammatory cells and small vessels were seen in Case 1. In Case 2, islands of tumor cells were surrounded by fibrous tissue and dense inflammatory cells. Microcystic spaces containing pink serous fluid and lined by the epithelioid cells were focally seen. Edematous areas, rather than myxoid stroma, contained few extracellular hyaline globules. There was no necrosis.

**Fig. 2:**
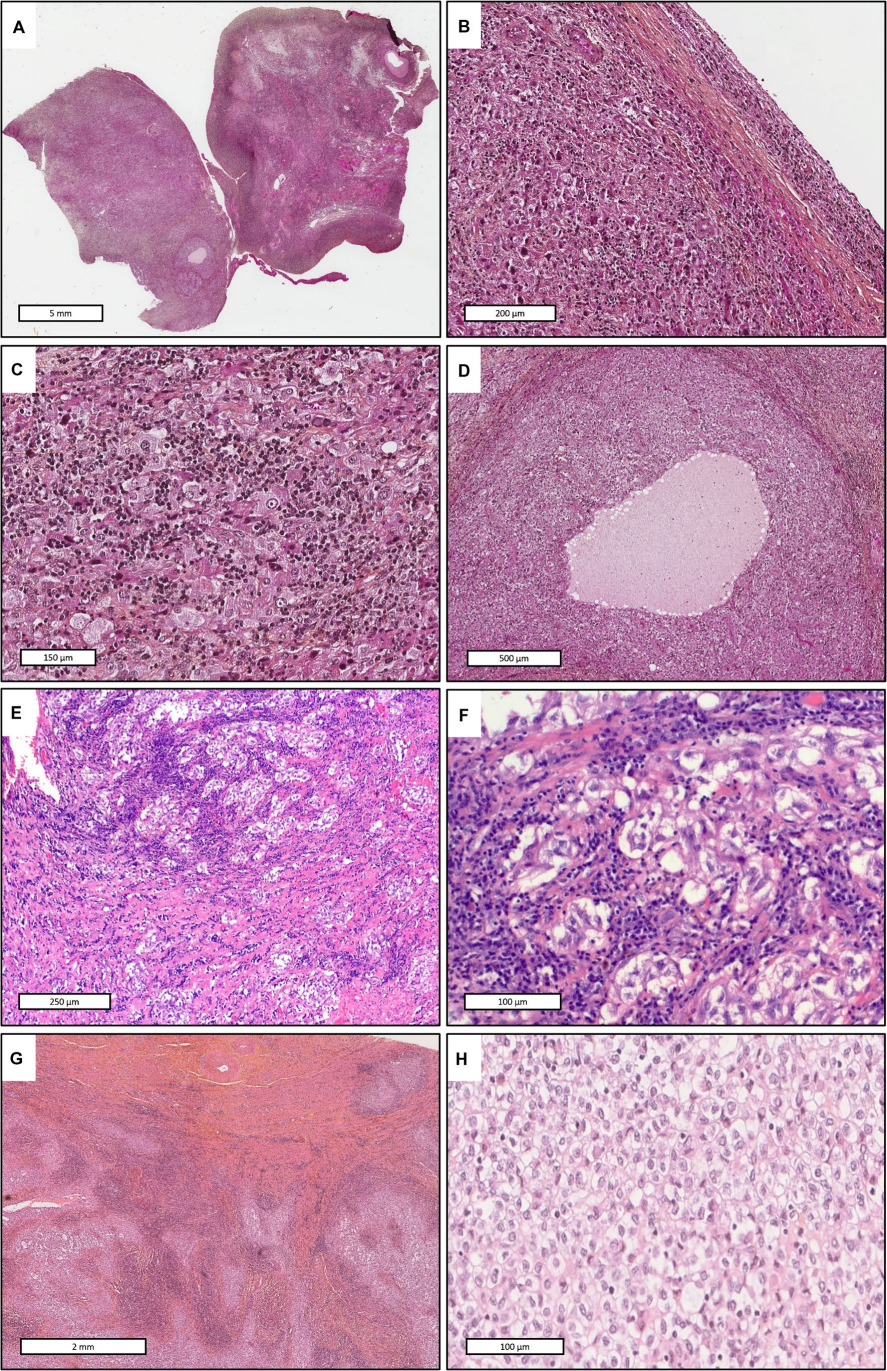
Histopathological features. Case 1 Hematoxylin-eosin-saffron (HES): **A** at low magnification, the tumor (left side) was attached to the ovarian surface (right side) and was surrounded by a thin fibrous capsule. **B** The tumor is well delimited by a thin fibrous capsule containing few inflammatory cells and covered by peritoneum. **C** Neoplastic cells are epithelioid with abundant eosinophilic cytoplasm, round nuclei containing vesicular chromatin. Cells are arranged in sheets, with a “syncytial-like” pattern and are surrounded by inflammatory infiltrate (lymphocytes, plasma cells). No papillary or glandular architecture is observed. **D** Microcystic spaces containing serous fluid and lined by the epithelioid cells are observed focally. Case 2 Hematoxylin and eosin (HE): **E** nests of tumor cells are separated by a fibrous stroma containing inflammatory cells**. F** Nests of epithelioid cells with clear or eosinophilic cytoplasm surrounded by dense lymphocytic infiltrate. Case 3 Hematoxylin-eosin-saffron (HES): **G** Tumor nodular extension into the myometrium. **H** Epithelioid cells with clear or eosinophilic cytoplasm growing in sheets

#### Immunohistochemical findings (Fig. 3)

All cases showed some degree of positivity with epithelial markers. EMA was focally positive in all three cases, while pankeratins AE1/AE3 and CAM5.2 were expressed only in Case 2. Desmin showed a diffuse and strong positivity in Case 1, a focal positivity in Case 2, but was negative in Case 3. MUC4 was positive in the majority of cells in Case 2, in less than 2% of cells in Case 1 and was negative in Case 3. Anaplastic lymphoma kinase (ALK) protein showed a strong expression in Case 2 and 3 but was negative in Case 1. WT1 was focally positive in Case 1 and 2 and negative in Case 3. Synaptophysin and chromogranin were expressed in Case 2, only.

**Fig. 3:**
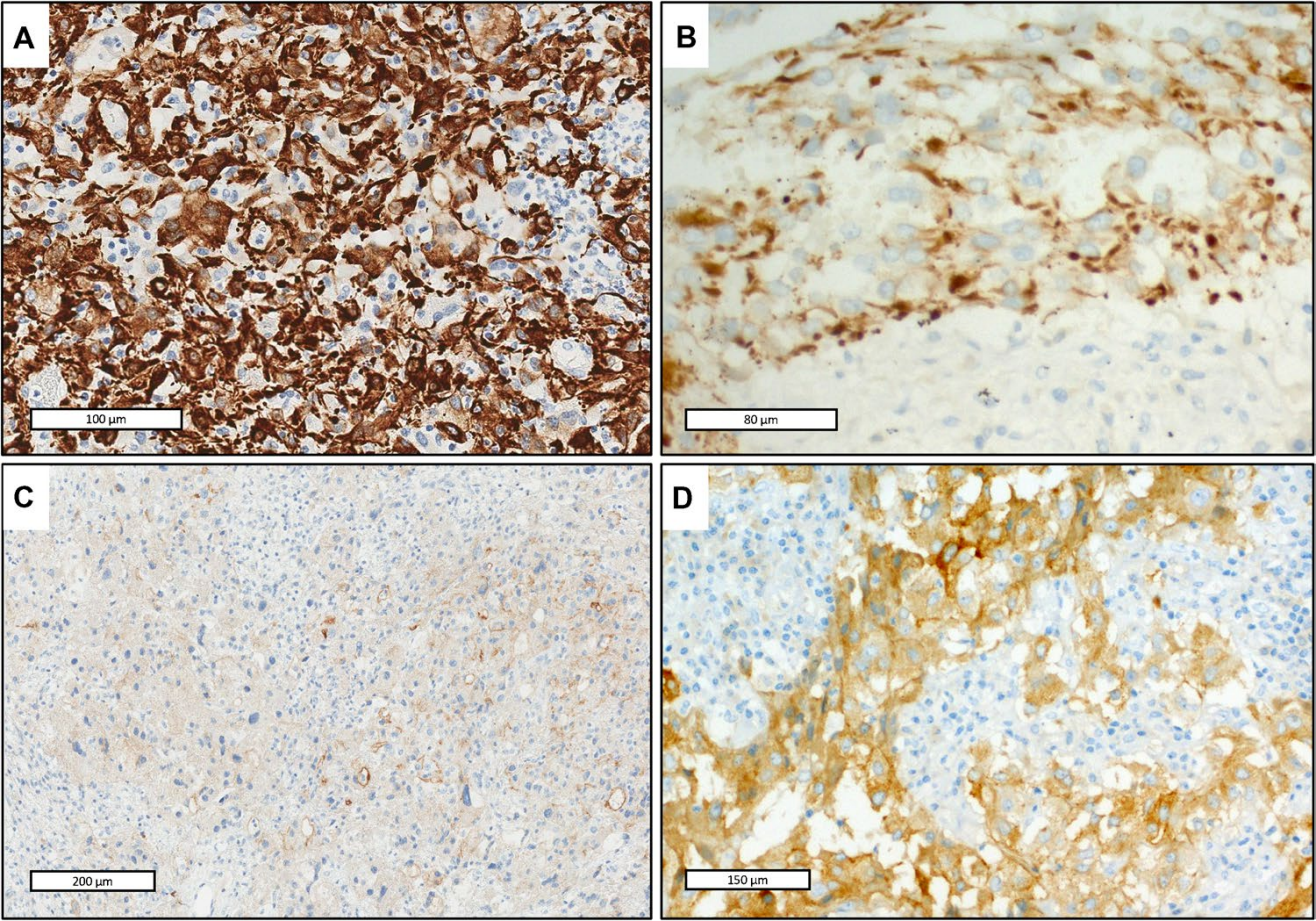
Immunohistochemical findings. **A** (Case 1, Immunoperoxidase): diffuse and strong positivity of tumor cells using anti-desmin antibody. **B** (Case 2, Immunoperoxidase): tumor cells express pancytokeratin AE1/AE3. **C** (Case 3, Immunoperoxidase): focal expression of EMA by the tumor cells. **D**. (Case 2, Immunoperoxidase): diffuse ALK protein positivity in tumor cells

All three cases were totally negative for the following markers: cytokeratin 7, CD34, CD117, DOG1, SALL4, HMB45, Melan A, SOX10, S100 protein, myogenin, estrogen, and progesterone receptors, PAX8, GATA3, GFAP, TFE3, CathepsinK, nuclear betacatenin, panTRK, inhibin, calretinin, SF1, and FOXL2. The expression of SMARCB1/INI1, SMARCA4/BRG1, and BAP1 was retained in all cases. P53 showed a wild pattern of staining.

### Molecular findings

RNA sequencing analysis revealed *EWSR1::ATF1* gene fusion in Case 1 and 2, with exon 6 of *EWSR1* fused to exon 4 of *ATF1.* An *EWSR1 (exon 15)::CREM (exon 7)* fusion was detected in Case 3.

Furthermore, exome-based RNA capture sequencing was performed to compare the expression profile of tumor 1 with more than 5000 other samples using clustering methods to confirm the fusion genes and small nucleotide variations, as previously described [11]. This analysis confirmed the high transcriptomic proximity of this tumor with the group of soft tissue AFH (data not shown).

## Discussion

The EWSR1/FUS-CREB fusion family of neoplasms have been ever expanding to encompass a variety of histologically, immunophenotypically, biologically, and topographically distinct entities. This was the consequence of the ever-increasing wide use of next generation sequencing tools in routine surgical pathology. With accumulating knowledge, it became evident that the mere presence of a specific gene fusion does not define an entity without consideration of other anatomic and immunophenotypic context. This is more obvious for clear cell sarcoma (definitionally with a melanocytic cell phenotype) and malignant gastrointestinal neuroectodermal tumor (coexpression of S100 and SOX10 but not specific melanocytic markers). Other entities in this genetic tumor spectrum such as AFH possess defining morphological (e.g., syncytial epithelioid or spindle cells in fascicular growth associated with variable aneurysmal/angiomatoid changes and lymphoid cuffs) and immunophenotypic (variable expression of cytoplasmic CD99, desmin, EMA, and ALK) features. Another subset is defined principally by prominent myxoid pattern mimicking extraskeletal myxoid chondrosarcoma and occurring at specific sites (lung, intracranial) but lacking specific immunophenotype. The last and most recently proposed member in this tumor category are intra-abdominal epithelioid neoplasms (sarcomas) that except for their frequent keratin expression, intra-abdominal location and neuroendocrine-like morphological and/or immunophenotypic features are not readily diagnosable by morphology and immunophenotyping alone. The latter group has been increasingly recognized in different abdominal sites.

CREB-associated sarcomas have not been defined in the gynecological tract, but they might have been under-recognized or diagnosed as other entities given their highly ambiguous and misleading immunophenotype as illustrated by our current cases. To our knowledge, only a single case of an AFH presenting as an ovarian mass has been reported to date. The tumor affected a 22-year-old woman, measured 6 cm in size and was resected 2 years after being incidentally discovered on ultrasound. The patient remained well, 8 months later. Molecular testing revealed an *EWSR1::CREB1* fusion [9]. Our study represents the first one to delineate these tumors at a novel anatomic site and at same time reporting features that are not AFH-typical in line with the emerging notion that these tumors might represent a distinctive family of neoplasms having the *EWSR1/FUS::CREB* fusions in common, but with variable morphological and immunophenotypic characteristics.

Two of our cases were associated with constitutional inflammatory symptoms such as fever, anemia, weight loss, and elevated serum CRP, all regressed immediately after tumor removal. Similar paraneoplastic phenomena are well known in AFH of soft tissue and are mostly mediated by IL-6 excess produced by the neoplastic cells. All of our cases and the one reported by Chen et al occurred in adult patients, 22 to 42 years old. The first ovarian case reported by Chen et al. [9] presented the morphology of AFH, with a characteristic pericapsular rim/ cuff of lymphoid tissue. Two of our cases were also surrounded by a fibrous capsule containing inflammatory cells but without aggregation of lymphocytes. However, our case 3 showed a malignant behavior with tumor extension into the myometrium and lymph node metastasis. Our cases showed variable morphological overlap with Chen et al.’s, being composed of islands of epithelioid cells separated by a sclerotic stroma infiltrated by chronic inflammatory cells. Bizarre and pleomorphic nuclei were seen without numerous mitoses. Based on the morphology and the immunoprofile with desmin and EMA positivity and the finding of an *EWSR1::CREB1* fusion, Chen et al. classified their case as an extrasomatic AFH [9].

AFH is a rare soft tissue neoplasm (0.3% of all soft tissue tumors) of low malignant potential (with 15% local recurrence and less than 2–5% metastases), typically occurring in the superficial soft tissues (deep dermis, or subcutis) of the extremities in children and young adults. It presents as slowly growing, superficial painless mass often simulating a hematoma [12]. Occasionally, systemic symptoms such as anemia, pyrexia, malaise, and weight loss, suggesting tumoral cytokine production, have been reported [12]. AFH lacks a specific immunophenotype, with desmin, EMA, ALK, CD68, and CD99 positivity being demonstrable in most cases [13]. In the appropriate morphological and clinical context, these markers proved valuable in recognizing AFH. In difficult cases, demonstration of the characteristic gene fusion involving *EWSR1* or *FUS* and one of the CREB family genes (*ATF1 or CREB1)* can be very helpful [13, 14].

Rare cases of AFH outside somatic soft tissue have been described involving the lung, mediastinum, bone, brain, vulva, retroperitoneum, and ovary [9]. As compared to their soft tissue counterparts, extrasomatic AFH occur in older patients (median age 35 versus 20-year-old), with a higher frequency of systemic symptoms (30% versus 10–15%), larger tumors, higher rate of recurrence (33% versus 11%), and a higher frequency of *ATF1* as fusion partner (63% versus 19%) [9]. The morphology of AFH is identical in soft tissue and extrasomatic site, with a characteristic peritumoral cuff of lymphoplasmacytic infiltrate, occasionally simulating a lymph node, a multinodular growth pattern, dendritic-like, and epithelioid tumor cells with eosinophilic cytoplasm and abundant admixed plasma cells [12]. Some cases present with a syncytial or histiocytoid aspect often admixed with abundant hemosiderin. We could argue that our two first cases, well-circumscribed, and showing *EWSR1::ATF1* fusion, could be closer to an AFH than to the CREM fusion family of malignant intra-abdominal epithelioid neoplasms. However, our case 3 demonstrated malignant features such as myometrial extension and lymph node metastasis, without any desmin positivity and had *CREM* as partner for *EWSR1*. Indeed, the majority of intra-abdominal malignant epithelioid neoplasms do not express desmin and involve *CREM* as partner for *ESWR1/FUS*, while AFHs show diffuse desmin positivity with *ESWR1/FUS::CREB1/ATF1* fusions, like our two first cases.

The location of our cases 1 and 3 at the serosal surface of the adnexa and the morphological findings reveal certain overlap with mesothelioma in young adults [5] that also occasionally harbors *ESWR1/FUS::CREB-*related fusions. Although epithelioid peritoneal mesotheliomas of young adults without a history of asbestos exposure may retain BAP1 expression and may lack calretinin positivity, they show at least focally some degree of frankly papillary or glandular patterns [5], features absent in our cases. In addition, our cases 1 and 3 lacked cytokeratin positivity which is required for the diagnosis of mesothelioma [5]. Overall, the morphology of the three tumors is not compatible with epithelioid mesothelioma.

In the female genital tract, an epithelioid neoplasm with numerous lymphocytes and plasma cells, such as that seen in our cases, suggests several differential diagnoses. A gestational trophoblastic tumor can be ruled out by the absence of diffuse keratin expression, while the absence of any immunoreactivity with H-caldesmon and hormone receptors is not in favor of an epithelioid smooth muscle tumor. Since 2/3 of AFH and epithelioid *EWSR1::CREB*-rearranged malignant neoplasms may express ALK protein, an epithelioid inflammatory myofibroblastic tumor (IMT) is a major differential diagnosis. However, the diffuse cytoplasmic ALK pattern is different from the distinct nuclear membrane ALK seen in majority of epithelioid IMTs. Moreover, lack of *ALK* and *ROS1* gene fusion by NGS excludes this possibility. Given the focal positivity of WT1, an adnexal sex cord stromal tumor is also in the differential diagnosis, especially since inhibin immunoreactivity has been described in *EWSR1/FUS::CREB*-rearranged tumors [6]. The expression of EMA in these tumors and our cases, along with the absence of hormone receptor positivity and of sex cord markers all argue against this diagnosis.

Parallel to our study, a series of male gonadal stromal tumors harboring *EWSR1::ATF1* gene fusions has been published online using the terminology “inflammatory and nested testicular sex cord tumor with aggressive clinical behavior and *EWSR1::ATF1* gene fusions” [15]. The morphology of these cases is quite similar to ours, including the presence of inflammatory cells and hyaline globules as well as the epithelioid aspect of the tumor cells, although nesting is more prominent in the testicular variant. Because of inhibin and SF-1 positivity and the nesting, these tumors have been designated as sex cord tumors. However, the positivity of EMA reported in all 9 testicular cases is very unusual for a sex cord tumor. Beside, inhibin positivity has been reported in *EWSR1/FUS::CREB*-rearranged tumors [6]. These testicular tumors might correspond to the gonadal male counterpart of the cases we report herein. However, all of our cases lacked sex cord marker positivity.

The most appropriate terminology for the cases we are reporting herein remains to be defined. We conclude that our cases do not represent variants of sex cord stromal tumors or any other defined mesenchymal/ stromal category of the female genital tract neoplasms. Instead, these tumors overlap significantly with the recently reported CREM fusion–associated intra-abdominal neoplasms, both morphologically and immunophenotypically, including occasional ambiguous (polyphenotypic) immunoprofile with expression of epithelial markers, MUC4, ALK, and neuroendocrine markers in subsets. In addition, frequent desmin reactivity and certain morphological features overlap with AFH of soft tissue. We propose the descriptive term “CREB fusion-associated epithelioid mesenchymal neoplasms of the female adnexa” for these tumors to enable better recognition by gynecological pathologists, and hence further characterization in the future. While the low number of reported cases does not allow for conclusive prognostic statement, it seems that these tumors of the adnexa likely are indolent but may be associated by locoregional aggressive behavior. As of now, none of the 4 reported cases developed distant metastases.

In conclusion, we described three cases of epithelioid neoplasms involving the female adnexa with *ESWR1::ATF1/CREM* fusions. These tumors belong to the spectrum of mesenchymal lesions harboring *ESWR1/FUS::CREB* family fusions involving the peritoneal cavity and intra-abdominal organs of young adults and including AFH-like and unclassified malignant epithelioid neoplasms. While AFH-like intra-abdominal neoplasms carry a low malignant potential, the CREB-positive intra-abdominal unclassified epithelioid neoplasms have a very aggressive behavior. As these tumors might feature a misleading bland or cystic appearance on imaging which combined with the young age may argue for a benign lesion, a high suspicion index and in particular presence of constitutional symptoms should alert to this differential. Same applies to their ambiguous and potentially misleading immunophenotype with a wide differential including sex cord tumors, mesothelial neoplasms, neuroendocrine tumors, IMT, and others. Accordingly, gynecopathologists should be aware of these entities’ in the differential diagnosis of an adnexal mass with epithelioid cell morphology.
